# Orthotopic bladder substitution: Surgical aspects and optimization of outcomes

**DOI:** 10.1002/bco2.84

**Published:** 2021-09-02

**Authors:** N. Thakare, B. W. Lamb, S. Biers

**Affiliations:** ^1^ Department of Urology Cambridge University Hospitals NHS Foundation Trust Addenbrooke’s Hospital Cambridge UK; ^2^ Faculty of Health, Education, Medicine and Social Care Anglia Ruskin University Chelmsford UK

**Keywords:** bladder substitution, cystectomy, neobladder, orthotopic bladder substitute, urinary diversion

## Abstract

**Objectives:**

Orthotopic bladder substitution (OBS) is a management option for urinary diversion in men and women undergoing cystectomy. The aim of the procedure is to provide a functional continent urinary reservoir of adequate capacity, compliance and low pressure. We have provided a narrative review of the existing literature and highlighted areas where improvement and standardization can be recommended.

**Methods:**

Literature search included database search for publications from January 1970 to November 2020, using keywords including OBS, bladder reconstruction, neobladder, radical cystectomy, robotic cystectomy, intracorporeal neobladder, surgical technique, patient selection and outcomes.

**Results:**

Due to various factors including indications, operative technique and risk of complications, OBS is an enormous undertaking and commitment for patients, surgeons and health professionals involved in the care pathway. The main considerations for patient selection, the technical elements of the procedure and the rationale behind these are discussed. Previously considered to be a choice for a select few, the inclusion criteria have expanded over the last decade. Similarly, surgical techniques including the choice and configuration of bowel segments, construction of anastomosis and nerve or organ sparing procedures have evolved over the years. Minimally invasive laparoscopic and robotic assisted surgery has added further perspectives to the existing literature on OBS. Understanding the principles of operative techniques and assessing the best evidence to influence patient management is crucial as it has a major impact on clinical outcomes. Peri‐ and post‐operative care, focused on the prevention of complications and morbidity, affects long‐term functional and oncological outcomes, which ultimately dictates the quality of life.

**Conclusions:**

This concise overview of OBS literature highlights the importance of pre‐operative, peri‐operative, and post‐operative aspects with regards to the optimization of patient care. To achieve the best results, meticulous attention should be paid in all these areas, surgical and multi‐disciplinary. Patient education and counseling, with shared decision making are central to the success of the procedure.

**Abbreviations**
FS: frozen section
ISC: intermittent self‐catheterization
LOS: length of stay
OBS: orthotopic bladder substitution
ONB: orthotopic neobladder
QoL: quality of life
RCT: randomized control trial
RC: radical cystectomy
RP: radical prostatectomy
UD: urinary diversion
WHO: World Health Organization

## 1 INTRODUCTION

The term orthotopic refers to a bladder substitute (neobladder) reconstructed in the same place as the native bladder, that is, in the pelvis. It is anastomosed to the native urethra, with a functional external urethra sphincter providing the continence mechanism. Orthotopic bladder substitution (OBS) was first described for male patients after radical cystectomy for cancer in 1913 by Lemoine using rectum.^1^ Small intestine neobladder was first reported by Camey and Le Duc in 1979.^2^ Use of an ileal segment is now the standard technique and radical cystectomy (RC) for bladder cancer is the main indication for OBS, which is less commonly performed for benign conditions including neurogenic bladder dysfunction.

In a consensus statement by the World Health Organization (WHO) and the Société Internationale d’Urologie (SIU), neobladder reconstruction was reported in 47% of patients undergoing radical cystectomy and urinary diversion.^3^ These figures were obtained from the institutions of the committee members from United States, Europe, Japan, and Egypt. This is highly disparate with the British Association of Urological Surgeons (BAUS) radical cystectomy audit 2014/2015, which showed only 5.7% of cystectomy patients underwent neobladder reconstruction.^4^ Similarly, recent reports from the United States National Cancer database show a declining trend toward continent diversion (12.1%) after radical cystectomy,^5^ likely due to the complexity of the procedure and concerns regarding the increased risk of complications with an increase in minimally invasive approaches.

Patients requiring RC are often referred from the center of initial diagnosis to a tertiary center for oncological management. An understanding of pre‐operative considerations, operative techniques, and post‐operative care is essential for the optimization of patients undergoing neobladder reconstruction. We outline the fundamentals of OBS primarily in the context of radical cystectomy. The essential steps to prevent pitfalls from the beginning of the patient journey and to achieve long‐term successful outcomes are discussed.

## 2 METHODS

A Medline database search using the following keyword search criteria was performed: OBS, bladder reconstruction, neobladder, radical cystectomy, robotic cystectomy, intracorporeal neobladder, surgical technique, patient selection, and outcomes. All publications from January 1970 to November 2020 were included and the literature search was confined to publications in English. An additional manual search of references in relevant published articles was performed.

## 3 PRE‐OPERATIVE PLANNING

Bladder substitution with intestinal segments is an option for continent diversion performed after cystectomy. The 2nd International Consultation on Bladder Cancer recommendations on the reconstructive options after RC (2012), have outlined criteria for patient selection with regards to renal function and risk of secondary tumor.^6^ Current EAU or AUA guidelines do not specifically offer recommendations for patient selection for OBS and much of the evidence has evolved over the years. Indications include cancer, chronic inflammatory disease (tuberculosis, schistosomiasis, post‐radiotherapy bladder contraction, bladder pain syndrome), and bladder dysfunction (neuropathic or idiopathic detrusor overactivity). OBS is very rarely performed for benign conditions and is mostly considered as a last resort when less invasive options have exhausted, in patients who wish to avoid an ileal conduit. The 2018‐2019 Hospital Episode Statistics (HES) database for NHS England reported a total of 2,165 ileal conduit and 486 urinary diversion operations were performed with cystectomy that year, and only 46 were simple cystectomy (benign) procedures.^7^

### 3.1 Oncological factors

For OBS following radical cystectomy, oncological aspects such as a complete resection of the primary tumor, negative margins, and the absence of metastatic disease are important. Pre‐operative transurethral biopsies from the prostatic urethra in males and bladder neck in females have a high negative predictive value, although studies show that they are not as accurate as intra‐operative frozen sections.^8^ Patients need clear counseling on the risk of developing secondary tumors in the urethra, which is 2.2% according to a systematic review by Fahmy et al.^9^ Recent studies show that patients with positive biopsies have an increased urethral recurrence rate, but the cancer‐specific survival is not reduced.^10^

### 3.2 Patient‐related

Patient profile is of utmost importance and careful consideration of co‐morbidities, performance status, life‐expectancy, and cognitive ability is needed. There is no age limit at which OBS can be offered, and it has been suggested that elderly fit patients have similar outcomes to their younger counterparts.^11^ There is no oncological compromise to women undergoing urethral‐sparing cystectomy.^12^ Adequate renal and hepatic function is an absolute prerequisite to reduce the risk of metabolic complications associated with the presence of bowel in the urinary tract. Previous significant bowel resection and severe inflammatory bowel disease predispose to the risks of Vitamin B12 deficiency, hyperoxaluria, and diarrhea, and hence are contra‐indications. Well‐informed motivated patients are ideal candidates as compliance with post‐operative bladder training and a follow‐up protocol is crucial.

### 3.3 Functional considerations

Functional abnormalities of lower urinary tract including urethral strictures should be excluded. External urethral sphincter dysfunction and stress urinary incontinence are relative contraindications. Pelvic irradiation is not considered an absolute contraindication; however, continence rates are lower (56%‐76%).^13^, ^14^ The risks of 90‐day post‐operative complications are higher (77% vs. 52%) in non‐neobladder UD with previous irradiation.^13^ Late sequelae such as bowel stenosis, spontaneous neobladder perforation, and neobladder‐vaginal fistula were seen in 40% of OBS patients in this study. Patients should be counseled that additional continence procedures may be required, with artificial urinary sphincter being placed in around 20% of such patients.^14^ Prior radical prostatectomy is not a contraindication in men, especially if they have good continence pre‐operatively.^15^

### 3.4 Other factors

All options for urinary diversion including ileal conduit should be discussed with the patient and shared decision‐making should be implemented. Involvement of a specialist nurse early on in the management pathway is essential. Initiation of the Enhanced Recovery Protocol for RC has shown to reduce median LOS from 14 days to 7 days in the UK^16^ and improved overall outcomes by earlier return to normality.^17^ We have summarized the pre‐operative factors to be considered prior to RC and OBS in Table 1.

**TABLE 1:** Factors to be considered for pre‐operative planning for orthotopic bladder substitution

Criteria		Comments
Oncological	Resectable disease No lymph node involvement No distant/lung metastases Prostatic urethral biopsies in men negative Bladder neck biopsies in women negative	Positive biopsies increase the risk of urethral recurrence, but patients can still be considered for OBS as survival is not affected Intra‐operative FS more accurate
Patient‐related	Age/life expectancy Renal function Hepatic function Bowel disease Cognitive function Motivation	Renal and liver impairment and bowel disease are absolute contra‐indications A motivated fit ‘elderly’ patient is still a candidate
Functional	Exclude urethral stricture No sphincter dysfunction No stress urinary incontinence (SUI) Previous radical prostatectomy (RP) Previous pelvic radiation	Sphincter dysfunction and SUI are relative contraindications Previous RP or radiotherapy is not contraindicated
Other	Enhanced Recovery Protocol Shared decision making Specialist nurse input	Desirable factors

## 4 KEY SURGICAL PRINCIPLES

The main principle is to provide a continent urinary reservoir of adequate capacity, compliance, and low pressure. The advantages are continence (and the avoidance of a stoma bag), potential for spontaneous voiding and improved body image.^18^

Careful dissection around the urethral margin to preserve the optimal length of urethra is necessary. Intra‐operative frozen section is recommended to confirm negative margins. Prostatic urethral tumors are associated with an increased risk of secondary urethral tumors (12%‐18%),^19^ and multifocal disease and carcinoma‐in‐situ (CIS) is also reported to increase the risk of prostatic urethral tumor and secondary urethral tumors. These factors were previously considered a contraindication to OBS, but studies show that they do not appear to significantly increase the risk of secondary tumor.^9^ If the distal urethral margin is negative intra‐operatively, OBS can be performed.^20^, ^21^ The incidence of urethral tumors is low in women undergoing cystectomy (1.4%), lower than for men (5%).^9^ Again, in appropriate cases, the suggestion is for frozen sections intra‐operatively, and if negative, proceeding with OBS.^22^, ^23^ If a frozen section shows tumor at bladder neck in women, the risk of developing urethral recurrence is 50%.^22^

### 4.1 Choice of the bowel segment

Ileum is the most commonly used bowel segment as it has better compliance and less contractility as compared with colon or cecum. Ileal neobladder can obtain the same capacity as a colonic neobladder, but ileal storage pressures are lower,^24^ and there is less reabsorption of components from the urine due to mucosal atrophy over time, as compared to colon. Other segments of the intestine that have been used include ileum with cecum and or colon (Mainz pouch, Le Bag), right colon (Indiana, Goldwasser), and sigmoid. A meta‐analysis of two trials suggested no difference in daytime or nocturnal continence rates between ileocolonic segments (using the Le Bag technique) or ileocaecal segments compared with and ileal segment (using the Studer technique).^25^, ^26^ Only one trial was identified that suggested ileal neobladder had lower rates of nocturnal incontinence, compared to ileocolonic segments,^26^, ^27^ the other study did not identify any differences.^25^

### 4.2 Techniques of bowel configuration

The chosen bowel segment is detubularized and the segments are reconfigured to create a spherical‐shaped reservoir. This obtains the maximal volume from the minimum absorptive surface area of bowel. Detubularization also lowers the reservoir pressure, reducing the risk of vesicoureteric reflux, and lowers the risk of spontaneous contractions causing urinary leak. However, it also increases the risk of requiring intermittent self‐catheterization (ISC). Examples of ileal neobladder include the Studer, Hautmann, Vescica ileale Padovana (VIP), Orthotopic Kock pouch (or hemi‐Kock), and Camey II neobladder. Table 2 gives a brief summary of the techniques used in bladder reconstruction. The Studer pouch (with various modifications) is the most commonly used technique as it is relatively easier to perform.

**TABLE 2:** Techniques of bladder reconstruction using bowel segments

Technique	Bowel segment	Principle
Studer	Ileum	60 cm terminal ileum isolated (25 cm away from ileocaecal valve); 40 cm used to form a reservoir; 20 cm used to form the afferent tubular limb for anastomosis of ureters (to prevent reflux)
Hautmann W Pouch	Ileum	70 cm of ileum incised along the antimesenteric border and arranged into a ‘W’ configuration. Ureters are reimplanted from inside the neobladder via a small incision and are non‐refluxing
Vescica ileale Padovana (VIP)	Ileum	40 cm segment of ileum is isolated (15‐20 cm away from the ileocaecal valve). The detubularized ileum is fashioned in a circular manner to create a spherical reservoir. The ureters are spatulated and passed through the posterior aspect
Orthotopic Kock Pouch	Ileum	60 cm ileum; the proximal 16cm is used for making the afferent nipple Two long segments are used to construct a ‘U’ shape, and an ileal plate is made. A mesenteric window is created at the proximal aspect of the afferent limb, which is intussuscepted onto the ileal plate and stapled in place. The ureters are stitched to the proximal part of the afferent limb with the Wallace technique
Camey II	Ileum	65 cm of ileum is isolated, detubularized, and configured unto a ‘U’ shape. The neobladder is attached to the urethra in the mid‐point and laterally fixed to the pelvic side wall. Non‐refluxing ureteric re‐implantation is performed into the lateral limbs of the reservoir
Mainz Pouch	Ileocolonic	10‐15 cm cecum and ascending colon and 20‐30 cm terminal ileum are isolated and detubularized, and anastomosed side‐to‐side to create a pouch
Indiana Pouch	Right colon	Detubularized right colon used

Note

Of note, the Koch nipple valve is no longer in common use because of an increased rate of complications.

### 4.3 Urethral and ureteric anastomoses

Anastomosis between the reconstructed bladder outlet and the urethra (preserving the sphincter) is created in a manner that it sits flat in the pelvis. A funnel‐shaped outlet is not favored as it has risk of kinking and mechanical obstruction. This is especially important in women as urethral‐pouch flexion can lead to chronic retention.^28^ As described by Studer, the reservoir can be held in place by anchoring sutures to the Denonviller's fascia and pubo‐prostatic ligaments. This allows for minimal tension on the membranous urethra and urethro‐pouch anastomosis. The ureters are mobilized, and implantation is achieved by an end‐to‐side anastomosis either directly into the reservoir or into an afferent tubular limb as described by Studer.^29^ Temporary ureteric catheters are inserted and brought to the abdominal wall to divert the urine and protect the anastomotic suture line from leaks.^30^

### 4.4 Anti‐reflux mechanism

The question of whether reflux in a low‐pressure reservoir such as OBS is clinically important has been debated. The WHO Consensus Group reported that reflux is not of clinical importance in OBS, but an anti‐reflux mechanism is essential in continent cutaneous pouches.^3^ Other studies indicated a trend toward an increased risk of stenosis (and subsequent upper tract deterioration) with anti‐refluxing versus freely refluxing uretero‐intestinal anastomotic techniques in OBS.^29^, ^31^ One study randomized ileal OBS patients to either anti‐refluxing nipple valve or an isoperistaltic afferent ileal tubular segment for reflux prevention.^29^ After a median 45‐57 months follow‐up, there was no difference in the incidence of urinary tract infection (UTI), urinary incontinence, serum creatinine or functional reservoir capacity between the two groups. However, stenosis and upper tract deterioration were higher with the anti‐refluxing technique (13.5% vs. 3% in the refluxing group).^29^ When strictures do occur, they are associated with renal impairment.^31^ Currently, oncological surgeons, who perform OBS, omit the formation of an anti‐refluxing mechanism.

### 4.5 Nerve sparing and organ sparing

Nerve‐sparing in men is performed using the same technique as radical prostatectomy by dissection of the plane between the prostatic capsule and the neurovascular bundle. Nerve sparing can be unilateral on the non‐tumor bearing side so that oncological safety is not compromised. In both male and female patients, preservation of autonomic innervation has a positive impact on continence with long term benefits.^32^, ^33^ Similarly, nerve sparing also prevents erectile dysfunction in men and loss of sexual function in women.^34^, ^35^ In women, genital sparing surgery is recommended as it is known to maintain continence and sexual function,^36^ although further studies are required to assess its benefit over standard RC.^37^ Preservation of the anterior vaginal wall has been shown to improve function, without compromising negative margins.^38^ Prostate capsule and seminal vesical preserving procedures were previously discouraged due to poorer functional and oncological outcomes.^39^, ^40^ Recent studies, however, suggest that in select cases prostate capsule sparing has shown better continence and erectile function rates, with similar oncological outcomes.^41^ In one recent study, functional outcomes, at 3 months after surgery, 90% of men reported daytime urinary continence in the prostate capsule sparing group versus 51% in the non‐sparing groups and 53% versus 9% patients showed erectile function recovery.^42^ A systematic review by Hernández et al, concluded that in men, sexual function preserving cystectomy including prostate‐, seminal vesicle‐ and nerve‐sparing showed higher potency rates.^43^

### 4.6 Minimally invasive techniques

With the advent of minimally invasive and robotic‐assisted surgery, techniques for ONB reconstruction have also shown advancement, though the basic principles remain the same. Robotic cystectomy and extracorporeal neobladder formation involve the construction of the ileal pouch in an open manner followed by urethro‐enteric anastomosis performed with the robot using the “Van Velthoven technique”.^44^ The Karolinska‐modified Studer neobladder technique, a total intracorporeal robotic neobladder reconstruction using Ligaloop bands was pioneered by Jonsson et al.^45^ Other intracorporeal robotic neobladders described in large series include the University of Southern California–modified Studer neobladder, the pyramid pouch, and the Y‐pouch.^46^ Whether intracorporeal approach has advantages in terms of post‐operative recovery, is not fully established, reports have shown a longer learning curve and slightly higher risk of complications compared to extracorporeal diversion.^47^, ^48^ The currently ongoing iROC trial is a prospective RCT comparing outcomes of robotic cystectomy and intracorporeal diversion with the outcomes of open RC.^49^ One study comparing open with robotic intracorporeal neobladders showed that short‐term results for urodynamics and Health‐related quality of life (HRQoL) score were similar, although daytime incontinence was worse for intracorporeal ONB.^50^ The authors ascribed this to the shorter period of post‐operative recovery in the robotic group.

**FIGURE 1:**
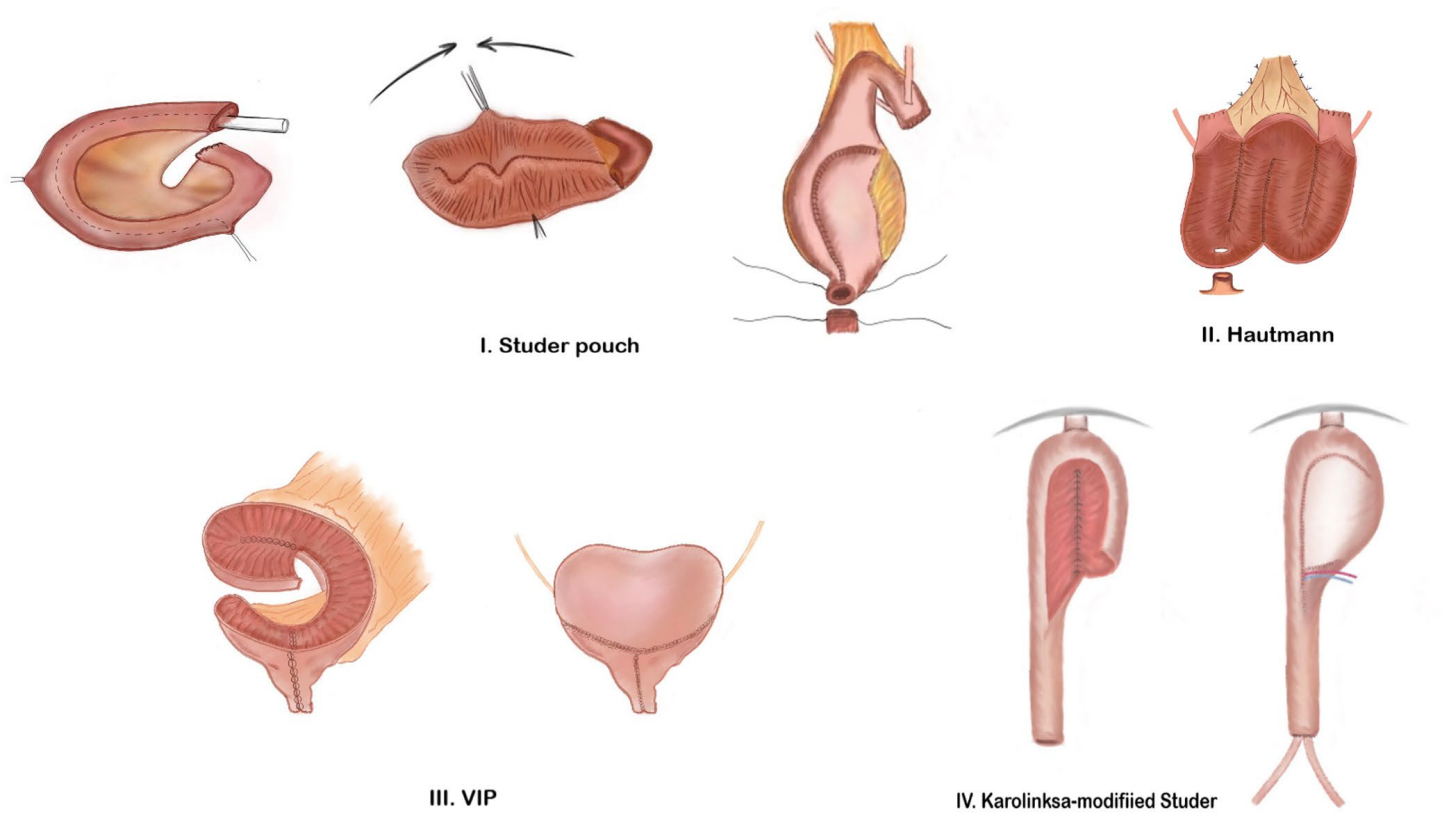
Diagrammatic representation of the commonly performed ileal neobladder variants. (I) Studer pouch, (II) Hautmann, (III) Vescica ileale Padovana (VIP) and (IV) Robotic intracorporeal Karolinska‐modified Studer pouch

Figure 1 shows illustrations of the commonly performed ileal neobladder variants including Studer, Hautmann, VIP, and the robotic intracorporeal Karolinska‐modified Studer pouch.

## 5 POST‐OPERATIVE CARE AND COMPLICATIONS

In the post‐operative period, management of catheters is important, with regular flushing and removal of mucus and blood clots from the neobladder. Removal of ureteric and suprapubic catheter depends upon institutional practice and there is no consensus on the placement or duration of catheters. As per Studer's original description,^51^ the ureteric catheters can be removed between 5 and 8 days post‐op and a cystogram performed at 8 to 10 days with removal of suprapubic catheter followed by urethral catheter removal 48 hours later. After the removal of catheters, patients are trained to void and undergo a period of bladder rehabilitation. Voiding is initiated by relaxing the sphincter and pelvic floor, while gently increasing intra‐abdominal pressure. Gradual increase in neobladder capacity is achieved by increasing the intervals between voids. Post‐void residuals are measured to ensure emptying although there are no strict cut‐offs for ideal residual volumes. Positive urine culture occurs in up to 10% of patients due to resident bowel flora. Incomplete emptying, excess mucous production and upper tract obstruction should be excluded and must be treated with antibiotics even if asymptomatic. A systematic review of urinary diversions showed that OBS had lower postoperative morbidity (14%) and mortality (1%) as compared to ileal conduit (21% morbidity and 2% mortality rate), although this did not reach statistical significance.^52^ It could be argued that these differences were due to selection bias involved in patients undergoing different types of urinary diversions. Overall early complications (within 30 days post‐operatively) in ileal neobladders are reported in around 23%.^53^ The MSKCC standardized reporting system^54^ defines 11 categories of post‐RC complications as summarized in Table 3.

**TABLE 3:** Summary of complications in patients undergoing Radical Cystectomy and Orthotopic Bladder Substitution

*Post‐operative/Early complications* ^a^	
Gastrointestinal (29%)	Ileus, small bowel obstruction, anastomotic bowel leak, constipation, diarrhea, GI bleeding
Infectious (25%)	UTI, urosepsis, sepsis, pyelonephritis, abscess
Wound (15%)	Infection, wound dehiscence
Genitourinary (11%)	Urinary leak, fistula, renal failure, hematuria, retention, ureteric obstruction
Cardiac (11%)	Myocardial infarction, heart failure, arrhythmia
Pulmonary (9%)	Chest infection, atelectasis, pleural effusion, pneumothorax
Bleeding (9%)	Wound hematoma, post‐operative bleed
Thromboembolic (8%)	DVT, PE
Neurological (5%)	CVA/TIA, seizures, delirium, peripheral neuropathy
Miscellaneous (3%)	Psychological, dermatitis, tendonitis, acidosis, lymphocele, dehydration
Surgical (1%)	Bowel injury, vascular injury, incisional hernia
*Long term/Late complications*	
Urinary	UTI (upper 4%‐14%; lower 9%‐45%)
	Urolithiasis (5%‐7%)
	Uretero‐intestinal stricture (5%‐11%)
Renal function	Gradual decline in eGFR (31%‐74%)
Metabolic	Metabolic acidosis 4.5% [hypokalaemic hyperchloremic in ileal and hypochloraemia in jejunal neobladder]
	Vitamin B12 deficiency (5%)
	Osteoporosis; osteomalacia and hyperphosphatemia^b^
	Hepatic dysfunction^b^
	Risk of fractures (21%)
Functional	Urinary Incontinence (12%‐15%)
	Urinary Retention/incomplete bladder emptying requiring ISC (0%‐21%; up to 50% in women)
	Erectile dysfunction (35%)
	Female sexual dysfunction (45%)

^a^ Early complications: within 0‐90 days as per MSKCC standardized reporting

^b^ No studies reporting %age risk.

## 6 FOLLOW‐UP AND LONG‐TERM MANAGEMENT

The aim of follow‐up is the early detection of metabolic complications, management of voiding and sexual dysfunction and surveillance for cancer recurrence. There are no specific guidelines for post‐OBS follow‐up, and most regimes are guided by radical cystectomy follow‐up protocols as per the published EAU guidelines.^55^ Generally, a combination of urine culture (for UTIs), bloods tests (to assess for renal, hepatic function, and metabolic complications), post‐void residuals, upper tract imaging, voiding diary, and questionnaires is advocated.

Late complications (summarized in Table 3) are reported in 13% at a median follow‐up of 48 months, and in 31% at a median of 88 months follow‐up.^56^ A Cochrane Review reported no statistical difference in the incidence of UTI, uretero‐intestinal stenosis, and renal deterioration between OBS and other forms of urinary diversion.^27^ OBS is reported to have a median rate of lower UTI of 9%‐45%; upper UTI in 4%‐14%; requirement for ISC in 9%‐10%; urolithiasis in 5%‐7%; Vitamin B12 deficiency in 5%; diarrhea in 1% and re‐operation rate in 7%‐8%. The incidence of uretero‐intestinal stricture is between 5% and 11%.^51^ Other reported complications include mucus production, neobladder, and upper urinary tract stones, vaginal‐neobladder fistula, and OBS rupture.

Declining renal function can occur after OBS with 31%, 57%, and 74% risk of deterioration over 1, 5, and 10 years of follow‐up, which is similar to ileal conduit diversion and can be reduced by identifying modifiable factors such as post‐operative pyelonephritis, hydronephrosis and uretero‐enteric anastomotic stricture.^56^ Metabolic disturbances are dependent on the types of bowel segment used. Long‐term studies suggest a risk of around 4.5%.^57^ Ileum and colon can produce a hyperchloremic metabolic acidosis; jejunum can produce a hypochloremic, hyperkalemic metabolic acidosis and is less commonly utilized. While the majority do not need active treatment for this, sequelae of acidosis may include other electrolyte abnormalities, altered hepatic metabolism, osteomalacia, osteoporosis, urolithiasis, altered drug metabolism. Severe acidosis can lead to lethargy, vomiting and can be treated with fluids and alkalinization therapy with sodium bicarbonate for small bowel reservoirs and potassium citrate for large bowel reservoirs. Refractory cases of acidosis may be treated with chloride transport blockers (such as chlorpromazine). Chronic metabolic acidosis and bone loss can result in a 21% increased risk of fractures in RC patients.^58^

### 6.1 Functional outcomes

From a reconstructive aspect, a focus on functional outcomes is necessary as continence and sexual function contribute hugely to quality of life. Across several studies, continence is not uniformly defined, and therefore continence rates are difficult to quantify mainly because of the heterogenicity of data. Overall continence rates are reported at around 85%.^59^ Daytime continence rates are reported to be between 89% and 99%.^53^, ^60^, ^61^, ^62^, ^63^, ^64^ As well as continued bladder rehabilitation to increase neobladder capacity, pelvic floor rehabilitation is recommended in the form of pelvic floor muscle training supervised by a specialist nurse or physiotherapist. Despite a satisfactory operative technique and peri‐operative care, incontinence can be caused by overactivity in neobladders. Although the exact mechanism of overactivity is not known, pharmacological treatments including anticholinergics, imipramine, and intravesical Botulinum Toxin A have been tried.^65^ Nocturnal continence takes longer to return, up to 24 months, and is reported to affect 74%‐83%.^53^, ^60^, ^61^, ^62^, ^63^, ^64^ Nocturnal incontinence occurs due to the absence of the physiological detrusor sphincter reflex, decreased tone of urethral sphincter, and uninhibited neobladder contractions at night.^66^ Verapamil and oxybutynin have been shown to improve nocturnal continence rates.^67^ Continence rates after robotic intracorporeal neobladder construction are similar with up to 88% overall continence in one series^68^ and 87% daytime with 80% nocturnal continence in another large series.^69^

Continence does appear to be maintained in the longer‐term. In a study including three centers all performing ileal neobladders (Studer, Hautmann W Pouch or T pouch), at a mean follow‐up of 48 months, daytime and nocturnal continence rates were 99% and 78%, respectively.^63^ In the same patient group, follow‐up for a mean of 88 months, 98% of patients were still achieving daytime continence and 76% were maintaining nocturnal continence.^53^ Maintenance of continence in women after a median of 6.1 years follow‐up remained good with 82.4% daytime continence and 76.5% nocturnal continence.^70^ Of note, 58% of women in this study required periodic ISC. Women fare slightly less well overall than men, with daytime continence rates around 72%‐87%, and nocturnal continence around 66%‐85%.^12^, ^23^, ^38^, ^70^, ^71^ Complete continence is reported in 57%, of the remainder using pads, 66% used 1‐2 pads per day.^23^

The 2012 Cochrane review of urinary diversion and bladder reconstruction identified studies indicating 0%‐70% of patients who require ISC, while the remainder are able to empty the neobladder by abdominal straining.^27^ Rates of ISC for the Studer neobladder are around 0%‐21%.^53^, ^62^, ^72^, ^73^ In women, urinary retention (requiring ISC) is reported in 25%‐50%.^12^, ^23^, ^60^, ^74^, ^75^

Rates of erectile dysfunction in men without nerve‐sparing have been reported to be around 35% at 12 months follow‐up and are higher than that for ileal conduit (9.8%).^76^ Women commonly encounter sexual dysfunction with reported dyspareunia (22%), reduced libido (37%), difficulty achieving orgasm (45%), and decreased lubrication (41%).^77^ Reported studies show mixed results and as discussed above nerve‐sparing and organ‐sparing are beneficial.

### 6.2 Oncological outcomes

From an oncological perspective, there is no specific schedule recommended for follow‐up due to the limited data on surveillance.^78^ Recurrence is treated with urethrectomy and either ileal conduit or further reconstruction. Reconstruction involves closure of bladder neck and formation of a continent catheterizable channel via a cutaneous stoma. CIS may be treated with intraurethral BCG perfusion therapy.^79^ The risk of urethral tumor recurrence is lower for OBS than for cutaneous diversions. A recent systematic review showed that the risk of urethral recurrence was 2.2% with ONB versus 5.5% in non‐ONB urinary diversion.^9^ The authors concluded that muscle invasion, CIS, and prostatic stromal or urethral involvement at the time of RC have no significant effect on recurrence. There is some evidence that in male patients with symptomatic urethral recurrence, the survival rate is lower than those with no symptoms.^80^ EAU guidelines suggest that recurrence is more common within the first few years and recommend 6 monthly CT scans for 3 years followed by annual imaging. The Randomized Open versus Robotic Cystectomy (RAZOR) trial demonstrated that robotic RC is non‐inferior to open RC in terms of oncological outcomes.^81^ All urinary diversions in this trial were extracorporeal, performed using an open technique.

## 7 QUALITY OF LIFE (QOL)

Overall QoL remains good in most patients with OBS, with few differences between different types of diversion.^3^, ^82^, ^83^, ^84^ Health‐related quality of life (HRQoL) tested by the Short Form‐36 and a functional index questionnaire comparing orthotopic substitution with ileal conduit after radical cystectomy were favorable in both groups after a median follow‐up of 12‐15 months.^59^ While OBS patients had overall higher (favorable) mean scores for most parameters, there was no significant difference in most QoL indices, but issues with regards to body image were persistent in a patient with ileal conduit diversion. Ileal neobladder (Studer) patients had a significantly better physical function and a more active lifestyle (96% in OBS vs. 68% in the ileal conduit group).^59^

A few studies have shown an increased benefit in QoL specifically with OBS,^18^, ^85^, ^86^ with significantly better adaptation, self‐confidence and rehabilitation compared to ileal conduit, and improved return to normal activities. Patients who had OBS are more likely to recommended it as the preferred type of urinary diversion (97% vs 36% of conduit patients). Some report greater concern from ileal conduit patients regarding urinary leakage and adversely altered body image,^87^ and around 48.5% of patients had wet clothes due to urinary leakage during the day, versus 1.5% of neobladder patients.^18^, ^85^ Male patients having undergone OBS, have shown improved libido (47% vs. 21%) and erections as compared to ileal conduit (42% vs. 25%), although both groups reported similar low satisfactory sexual intercourse.^59^

At medium‐term (5‐year) follow‐up (mean 89 months), general health QoL was generally well maintained as compared to the “normal” population, unless patients required ISC or suffered from daytime incontinence.^88^ Studies reviewing QoL at 8.3 years follow‐up found no difference between ileal conduit and continent diversion although QoL scores tend to be lower for older patients, particularly over the age of 80 years.^89^

Several studies have assessed and compared the quality of life in patients undergoing different types of urinary diversion. Validated questionnaires such as EORTC QLQ‐30C, SF‐36, and FACT(‐BL) (‐G) (‐VCI) do not necessarily focus on the type of urinary diversion or specific functional outcomes and sexual function. Health‐related quality of life (HRQoL) was previously reported to be only marginally better with OBS compared to Ileal conduit diversion.^90^ A further meta‐analysis of non‐RCTs showed a clear benefit of OBS over ileal conduit in terms of HRQoL.^91^ The impact on the quality of life can also be assessed by the level of regret experienced after undergoing a certain type of diversion. Recent evidence comparing OBS with ileal conduit shows that shared decision‐making and goal concordance is necessary to achieve a low level of decision regret.^92^ Thus, is it vital that treatment outcomes should be discussed with patients and the choice to proceed with OBS made in an informed manner.

## 8 CONCLUSIONS

OBS is a valuable surgical option with the possibility of restoring the lower urinary tract function to achieve continence. This is important for patients undergoing RC as the “loss of an organ” is somewhat compensated by the psychological benefit of a “new one.” Over the years, indications for OBS have been refined and if strategically organized, the interplay between operative techniques and post‐operative care can lead to the most successful results. Further research is required in areas of minimally invasive techniques and standardization of long‐term management. Patients should be appropriately counseled regarding the long‐term outcomes and impact on the quality of life. Above all, the type of urinary diversion should be acceptable to the patient whose main concern is cancer recurrence.

## CONFLICT OF INTEREST

None.
